# Supplementary material for: Hybrid molecular graphene transistor as an operando and optoelectronic platform
Source: Nat Commun. 2023 Mar 13;14:1381. doi: 10.1038/s41467-023-36714-7 (PMC10011542; doi:10.1038/s41467-023-36714-7)
Supplement: Supplementary file 1 — Supplementary Information [file 41467_2023_36714_MOESM1_ESM.pdf]

Supplementary Information

Hybrid molecular graphene transistor as an operando and optoelectronic platform

Jorge Trasobares[1]✉, Juan Carlos Martín-Romano[1], Muhammad Waqas Khaliq[2], Sandra Ruiz-Gómez[2], Michael Foerster[2], Miguel Ángel Niño[2], Patricia Pedraz[1], Yannick. J. Dappe[3], Marina Calero de Ory[4], Julia García-Pérez[1], María Acebrón[1], Manuel Rodríguez Osorio[1], María Teresa Magaz[3], Alicia Gomez[3], Rodolfo Miranda[4,5], Daniel Granados[1]✉

SUPPLEMENTARY NOTE 1. ELECTROCHEMISTRY 2

SUPPLEMENTARY NOTE 2. RAMAN SPECTROSCOPY AND OPTICAL IMAGE 6

SUPPLEMENTARY NOTE 3. X-RAY PHOTOELECTRON SPECTROSCOPY 7

SUPPLEMENTARY NOTE 4. BAND DIAGRAM 10

SUPPLEMENTARY NOTE 5. DENSITY FUNCTIONAL THEORY 11

SUPPLEMENTARY NOTE 6. PLATFORM FABRICATION: OPTICAL, AFM AND CAFM IMAGES OF M-GFET DEVICE 13

SUPPLEMENTARY NOTE 7. ELECTRICAL TRANSPORT CHARACTERISTICS 15

SUPPLEMENTARY NOTE 8. M-GFET ELECTRICAL CONFIGURATION 21

SUPPLEMENTARY NOTE 9. ULTRAVIOLET PHOTOELECTRON SPECTROSCOPY, X-RAY ABSORPTION SPECTROSCOPY, THRESHOLD PHOTOEMISSION ELECTRON MICROSCOPY. 22

SUPPLEMENTARY NOTE 10. PHOTO RESPONSE 24

SUPPLEMENTARY REFERENCES 25

Supplementary Note 1. Electrochemistry

The ferrocene group is used both in electrochemistry (programable electrochemical rectifier[1]) and molecular electronics (molecular diodes[2]). Cyclic Voltammetry (CV) is applied to quantify the molecular energy (E_H), surface coverage (Γ) and intermolecular coulombic interactions (φ), the shape of voltammograms is modulated by the molecular interactions[3–5] where different molecular organizations due to surface roughness may lead to multiple CV peaks[3,6]. Additionally, oxidation potentials are related with the ionization potentials and work function change ($\Delta\Phi$)[7,8].

Here the electrochemical characterization of the Au/SC_{11}Fc SAMs presents the representative one electron transfer anodic and cathodic waves. Considering an intermolecular distance of 0.8 Å and an ion pairing distance of 4.9 Å, we can extract an average value per molecule-molecule coulombic interaction of φ=18 meV (Equation 4 Theoretical Methods). E_H falls in the expected range[2] (280 mV vs AgAgCl reference electrode). The *FWHM* relates quasi-linearly with the coulombic molecular interactions[5,9,10]. It indicates uniform distribution of the Fc groups into the SAM. Γ, area per molecule, obtained from the charge involved in the process is 0.32 nm^2/molecule close to the accepted values often reported in the field for the "standing up" conformation[3].

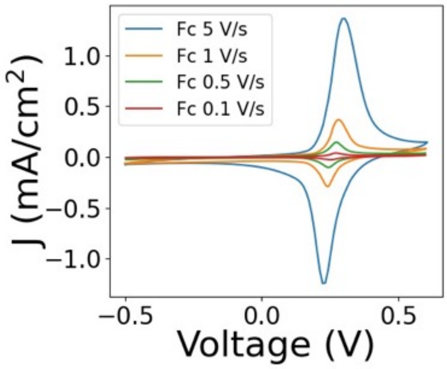

Supplementary Figure 1. Chemical characterization of Au/SC_{11}Fc. Cyclic voltammetry recorded on 6 mm diameter gold working electrodes at 0.1, 0.5, 1 and 5 V/s versus Ag/AgCl reference electrode, 1 M $NaClO_4$ electrolyte and a silver bar counter electrode.

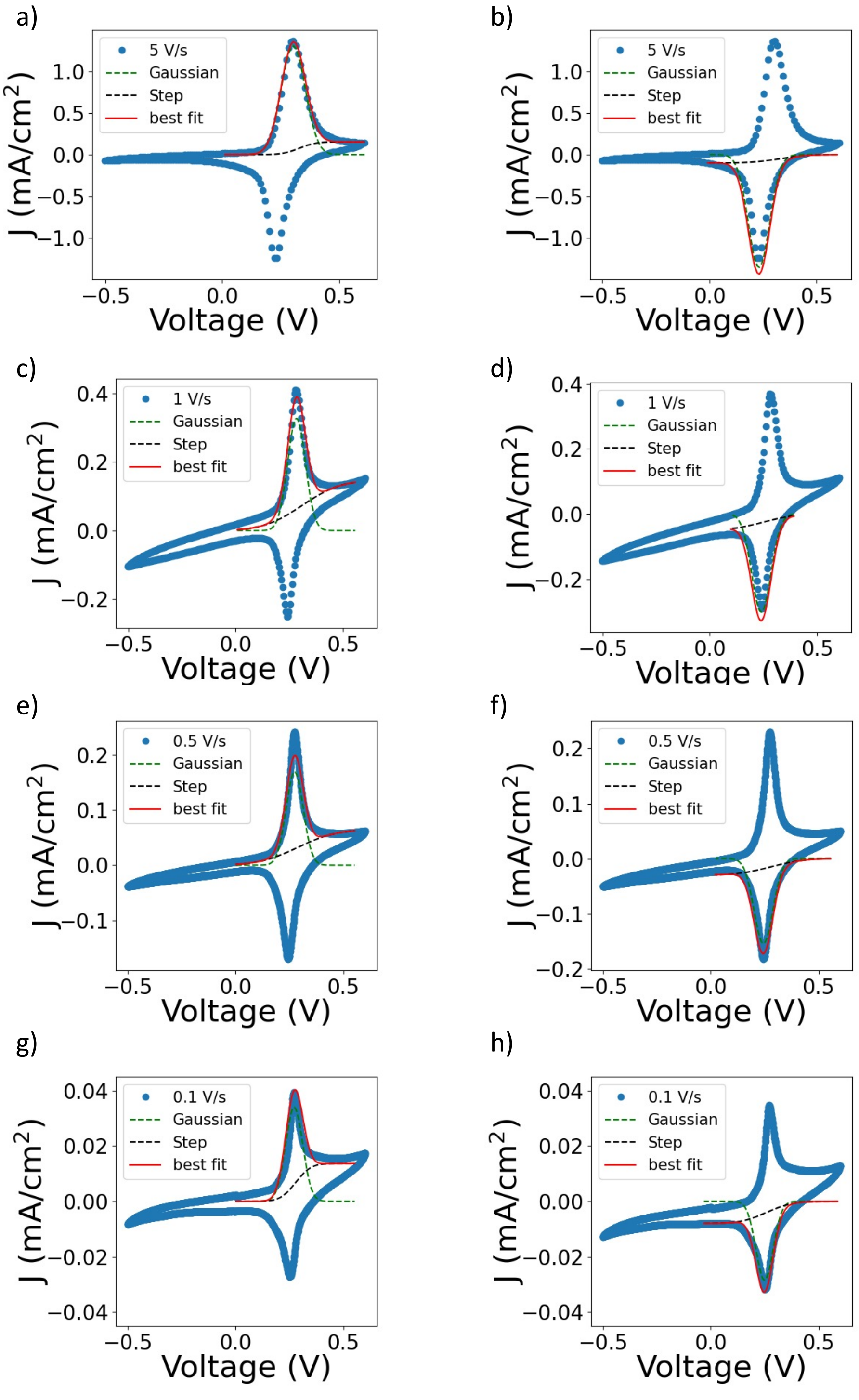

Supplementary Figure 2. Fitting of the electrochemical waves for Au/$SC_{11}Fc$ SAMs at different scan rates. (a,c,e,g) anodic peak at 5, 1, 0.5 and 0.1 V/s, **(b, d, f, h)** cathodic peak at 5, 1, 0.5, 0.1V/s versus Ag/AgCl reference electrode, 1 M $NaClO_4$ electrolyte and a silver bar counter electrode. Fits are obtained using a gaussian and a step functions with Non-Linear Least-Squares Minimization and Curve-Fitting for Python.

Au/SC11Fc//Gr SAMs reveals the oxidation and reduction waves appearing at the same energy level.

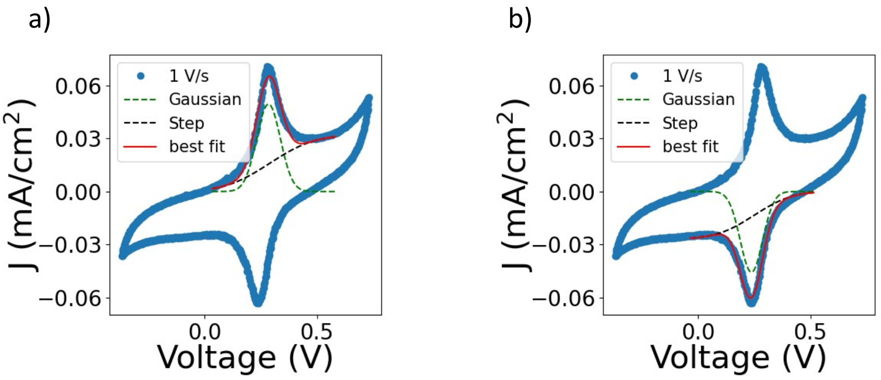

Supplementary Figure 3. Fitting of the electrochemical waves for Au/SC$_{11}$Fc//Gr SAMs at 1 V/s. **(a)** anodic peak, **(b)** cathodic peak versus Ag/AgCl reference electrode, 1 M $NaClO_4$ electrolyte and a silver bar counter electrode. Fits are obtained using a gaussian and a step functions with Non-Linear Least-Squares Minimization and Curve-Fitting for Python.

Supplementary Table 1. Electrochemical waves fitting parameters.

Anodic peak							
		**Au/SC_{11}Fc**					**Au/SC_{11}Fc//Gr**
**Scan rate** (V/s)		**5**	**1**	**0.5**	**0.1**		**1**
**Step**	Amplitude (C)	0.15	0.14	0.06	0.02		0.032
	Center (V)	0.33	0.31	0.29	0.28		0.29
	Sigma	0.08	0.20	0.20	0.08		0.22
**Gaussian**	Amplitude (C)	0.17	0.034	0.017	0.004		0.006
	Center (V)	0.30	0.29	0.28	0.28		0.29
	Sigma	0.05	0.04	0.04	0.06		0.05

Cathodic peak							
		**Au/SC_{11}Fc**					**Au/SC_{11}Fc//Gr**
**Scan rate** (V/s)		**5**	**1**	**0.5**	**0.1**		**1**
**Step**	Amplitude (C)	0.10	0.05	0.03	0.01		0.026
	Center (V)	0.33	0.25	0.28	0.26		0.25
	Sigma	0.15	0.16	0.17	0.13		0.18
**Gaussian**	Amplitude (C)	0.17	0.034	0.017	0.003		0.005
	Center (V)	0.23	0.25	0.26	0.27		0.23
	Sigma	0.15	0.16	0.03	0.03		0.05

Supplementary Note 2. Raman spectroscopy and optical image

Optical images and Raman spectroscopy demonstrate the formation of high-quality single layer graphene.

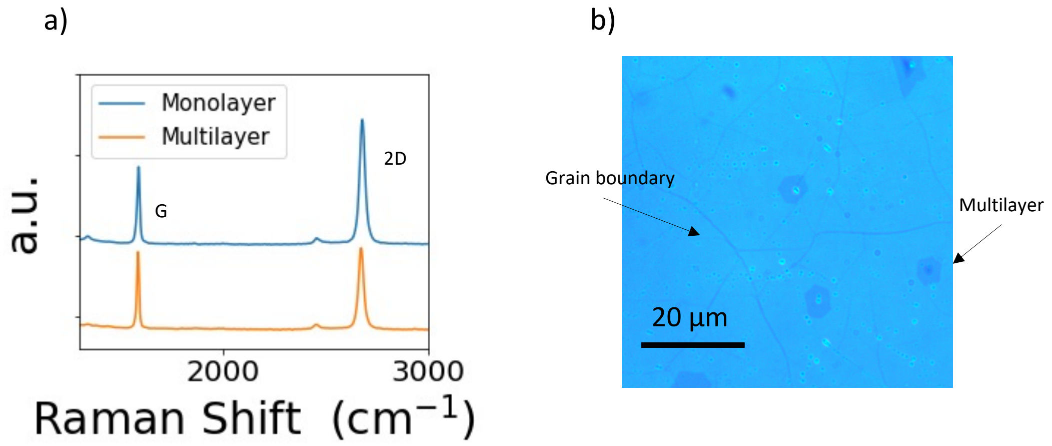

Supplementary Figure 4. Optical and Raman spectroscopy characterization of a CVD graphene layer transferred on a silicon wafer. (**a**) Raman spectra from single and multilayer zones. **(b)** Optical image of the CVD grown graphene.

Supplementary Note 3. X-ray photoelectron spectroscopy

In Supplementary Figure 5a, two peaks can be seen, located at E_b = 163.0 eV and E_b = 161.8 eV which are assigned to S $2p_{1/2}$ and S $2p_{3/2}$ core levels, respectively while in Supplementary Figure 5b the values are almost the same, E_b = 163.0 eV and E_b = 161.7 eV. These peaks fall slightly below the value of a S thiol bonded to Au (162.5 eV for S 2p 3/2). The study that agrees with earlier reports[11–14] includes fresh and aged (60 days at ambient conditions) samples to highlights the effect on the encapsulation over the time.

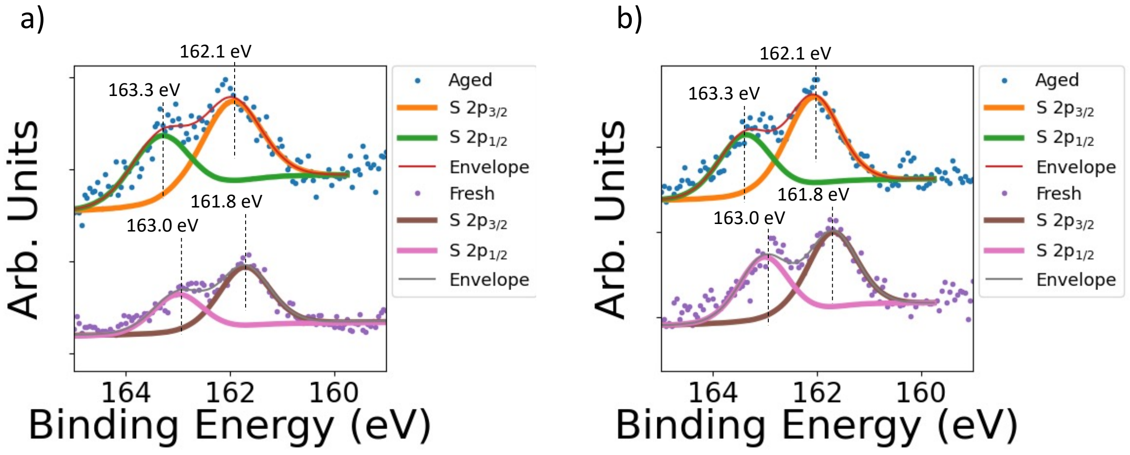

Supplementary Figure 5. XPS spectra of a ferrocenyl SAM samples, covered and uncovered by Gr. Core energy levels: **(a)** S *2p* on an uncoverd sample, **(b)** S *2p* in a sample covered with graphene before and after aging.

The XPS C *1s* spectra of the unprotected and graphene protected sample and its evolution with time are shown in the Supplementary Figure 6. For the unprotected ferrocene fresh sample (lower curve of Supplementary Figure 6a) the main peak is located at 284.6 eV, and we do not distinguish the different contributions of the carbon chain and the carbon in the ferrocene rings. There is some contamination, corresponding to C-OH or C-O components at 286 eV. For the fresh graphene protected sample (lower curve Supplementary Figure 6b) the main peak appears at a similar binding energy of 284.7 eV. Some other peaks appear at 286, 287.7 y 289 eV, that are compatible with contamination and remaining residues of the transfer process[15]. For the aged samples (upper curves) we observe an increase of the carbon contamination peaks.

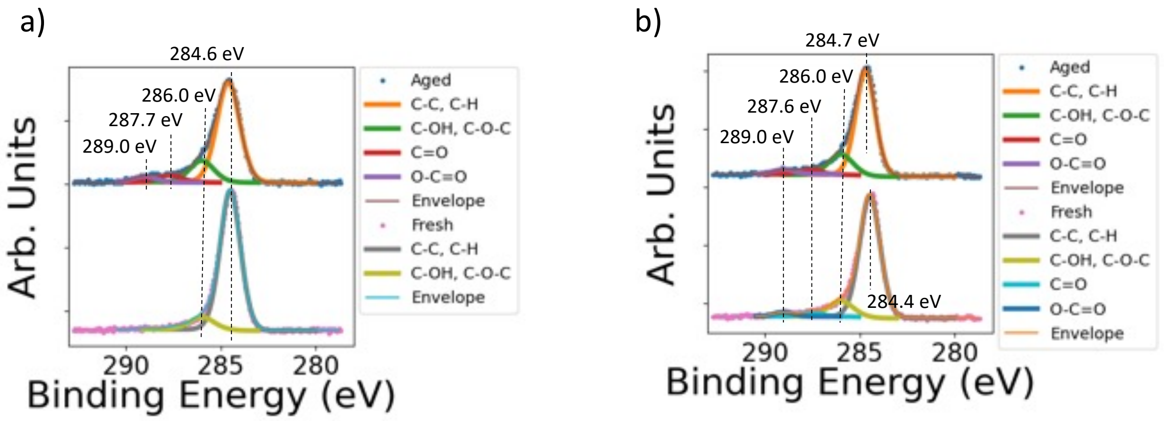

Supplementary Figure 6. C *1s* core level spectrum obtained from the measurement of **(a)** Au/FcC_{11} and **(b)** AuFcC_{11}//Gr before and after aging. The main peak of the spectrum, at 284.6 eV, encompasses both the peaks belonging to the ferrocene and the graphene, being both C-C bonds.

During operando measurements artificial shifts in the spectra proportional to the applied voltages are obtained both in the Fc *2p* and Au *4f* core levels. Additionally, a reversible decrease in the intensity while applying a voltage is observed for the Fc *2p* core level. This reduction is not observed in the Au *4f* core level intensities that remains unvarying independently of the applied voltage.

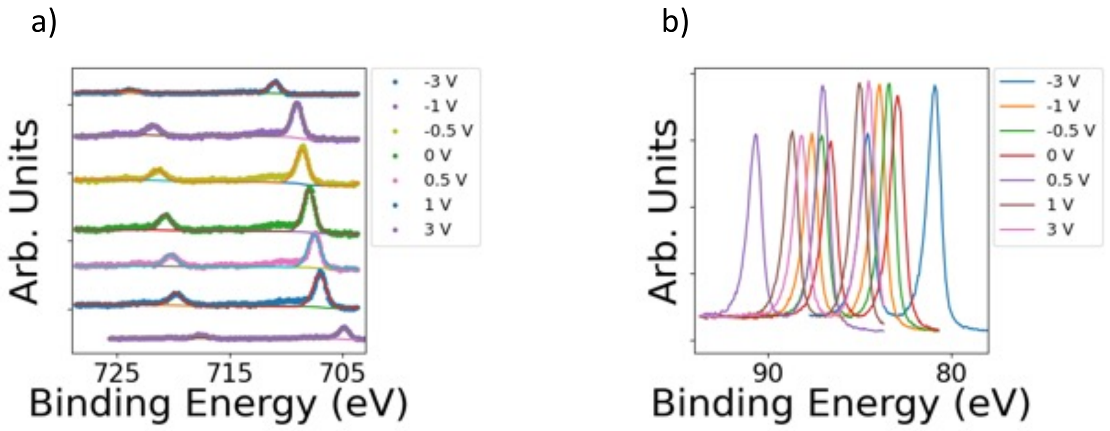

Supplementary Figure 7. XPS operando spectra. (a) XPS spectra of Fe *2p* core level of a Au/$SC_{11}Fc$//Gr sample to which a range of voltages was applied. **(b)** Au *4f* core level spectra of the same sample. Note that Binding Energy is artificially modified by the applied voltage.

Ultraviolet photoemission spectroscopy is applied to determine the work function of the surfaces. By measuring the full width of the photoelectron spectrum and subtract the photon energy we extract work function of: Φ_{Au} = 5 eV and $\Phi_{Au/SC11Fc}$ = 4.5 eV.

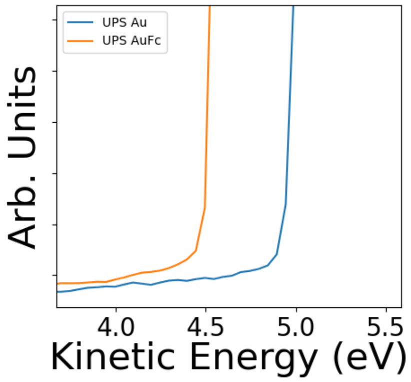

Supplementary Figure 8. UPS spectra of Au electrode, Au/$SC_{11}Fc$, and Au/$SC_{11}Fc$//Gr electrodes at the Kinetic Energy window of 4 to 6 eV.

Supplementary Note 4. Band diagram

When the device is at equilibrium, the Fermi level (E_F) is a constant (dotted flat line). In Supplementary Figure 9 the junction before and after the equilibrium are sketched for the theoretical values of the work function of Au and Gr. An electrostatic potential is generated due the differences in the work functions of the electrodes.

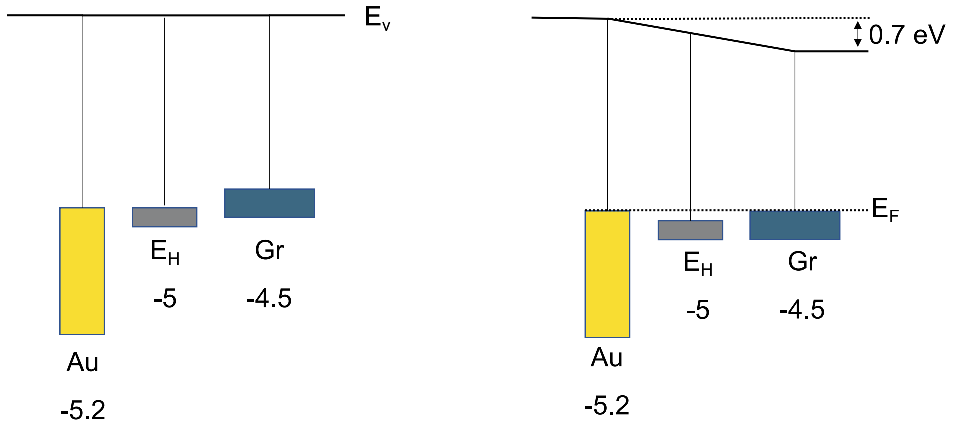

Supplementary Figure 9. Band diagrams of a gold/molecule/graphene junction. (a) Before equilibrium, **(b)** in equilibrium. Work functions are expressed in eV.

Supplementary Note 5. Density functional theory

The corresponding atomic configuration is represented in Supplementary Figure 10. Within a Density Functional Theory (DFT) formalism, we determine the electronic structure and in particular the projected Density of States (PDOS) of the molecule between the gold and graphene electrodes (Supplementary Figure 10b). As a result, we can notice a quasi-symmetric distribution of the electronic levels near the Fermi level (at around -0.5 and +0.35 eV). Such distribution let us anticipate a low rectification of the molecular junction, as well as a symmetric evolution of the electronic current when gating the system.

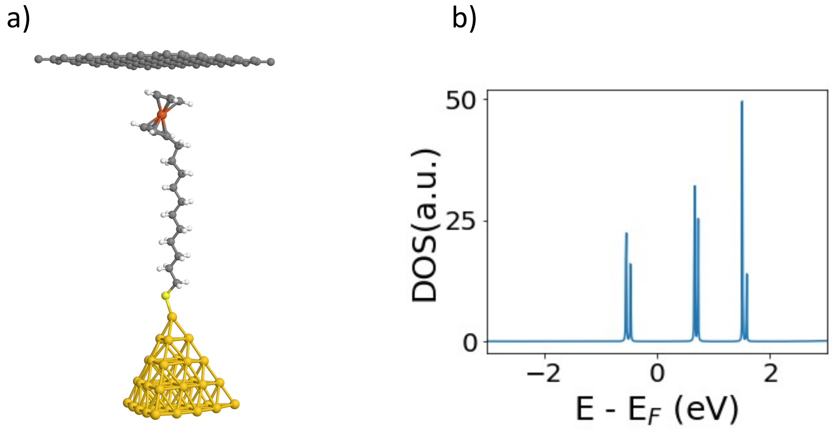

Supplementary Figure 10. Density Functional Theory. (a) Calculated representation of a single molecule junction, **(b)** Calculated Density of states of the system of the molecules in the molecular junction.

The electronic transmission of the junction as a function of the energy is presented in Supplementary Figure 11. The molecular junction with a graphene electrode presents a higher conductance than the junction with only gold electrodes. The introduction of graphene removes the rectification in the Fc junctions.

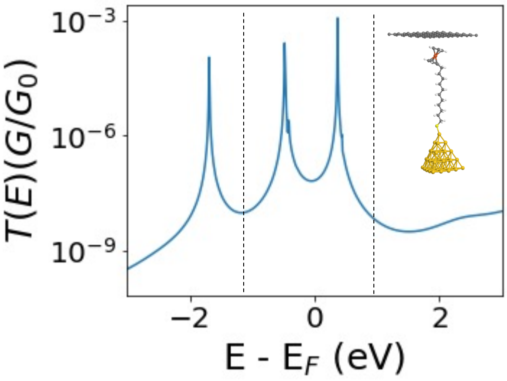

Supplementary Figure 11. Density Functional Theory. DFT calculated electronic transmission *T(E)* for the represented source-drain voltages. In inset, the atomic configuration of the model molecular junction used for the calculations.

We can notice from these calculations that the Dirac point (found in Γ due to the 3x3 unit cell) is very close below the Fermi level. That indicates there is no doping of graphene by ferrocene. Additionally, the total charge of graphene on Fc SAMs is almost equivalent to that of isolated graphene. This is easily explained by the π-π interactions between ferrocene and graphene that minimize energy level broadening[16].

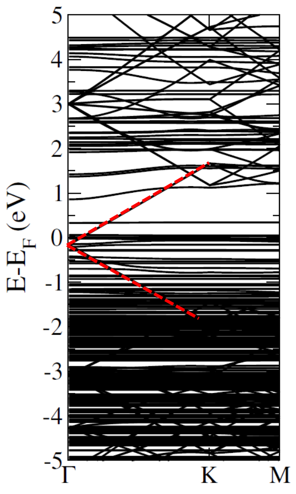

Supplementary Figure 12. Density Functional Theory. Au/SC_{11}Fc//Gr single molecule system band structure. Highlighted the Graphene Dirac point at Γ.

Supplementary Note 6. Platform fabrication: Optical, AFM and CAFM images of m-GFET device

Optical images are helpful to discern between single or multiple graphene monolayers as well as its quality. In Supplementary Figure 13a we can appreciate zones with high quality monolayer, bilayer and multilayers. Once the graphene is mounted in a m-GFET it is reshaped (highlighted by the dotted line in Supplementary Figure 13b). 2400 μm^2 of graphene is directly back gated between the silicon highly doped through the SiO_2. Note that 70% of the graphene is not screened by the electrodes. We have used 3 wafers (4 inch) containing 80 dies with 9 devices each die. Overall, 70.6% of devices results as the one in Supplementary Figure 13b. 17.1% presented some lithographic defects either on the electrodes or resin residues and 12.3% of the devices failed during the graphene transfer process.

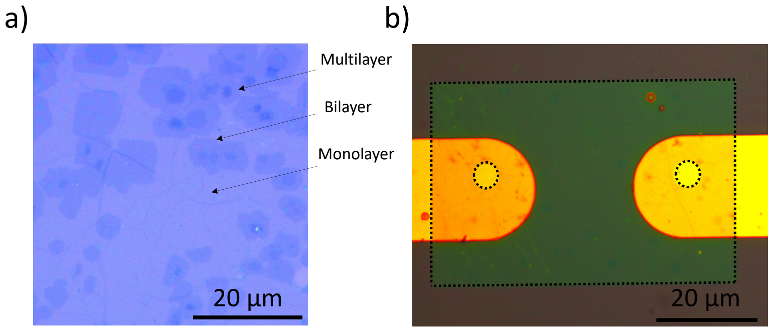

Supplementary Figure 13 Graphene optical images. (a) 50 x 50 µm image shows areas with multilayer that may play a role especially during the electron transfer. **(b)** Optical image of a m-GFET.

The topography study of the electrodes prior of molecular functionalization demonstrate that the HfO_2 etching is roughly 8 nm depth and the CAFM current versus voltage demonstrates an ohmic contact with the metallic electrode.

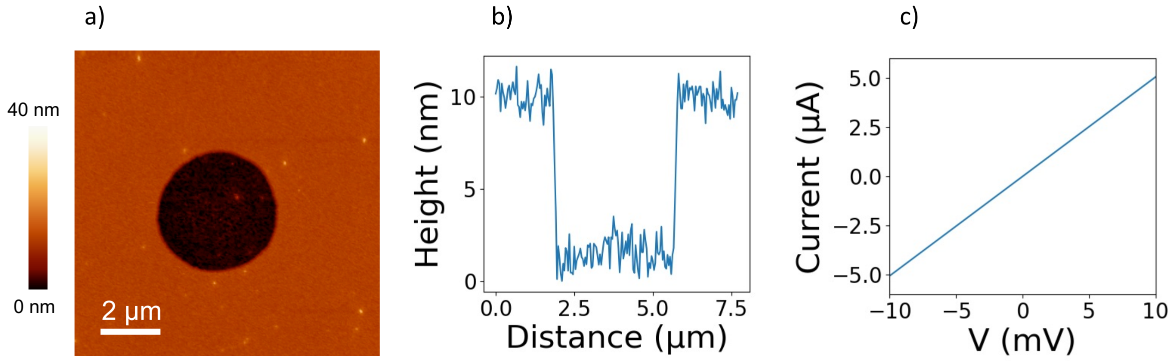

Supplementary Figure 14. Structural characterization of the electrode. (a) AFM image of the electrode. (**b)** Topographical cross section. (**c)** IV curve between the AFM conductive tip and the gold substrate in the etched zone.

Supplementary Note 7. Electrical transport characteristics

In our fabricated graphene field effect transistors (GFETs) the contact resistance is in order of KΩ while the graphene channel resistance is one order of magnitude lower.

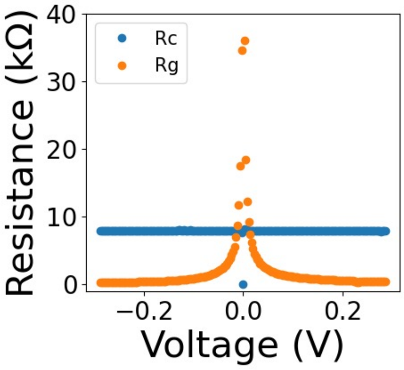

Supplementary Figure 15. Electrical characerization of the GFET. Contact and graphene channel resistance between source and drain in the G-FET.

A key feature in the GFETs is the type (holes and electrons) and density of carriers in the channel that can be controlled by the potential difference between the channel and the gate (V_{BG}). The situation where the semimetal graphene behavior changes from "hole conductivity" regime at the negative electrostatic potential to "electron conductivity" at the positive electrostatic potential is called the Dirac point[17].

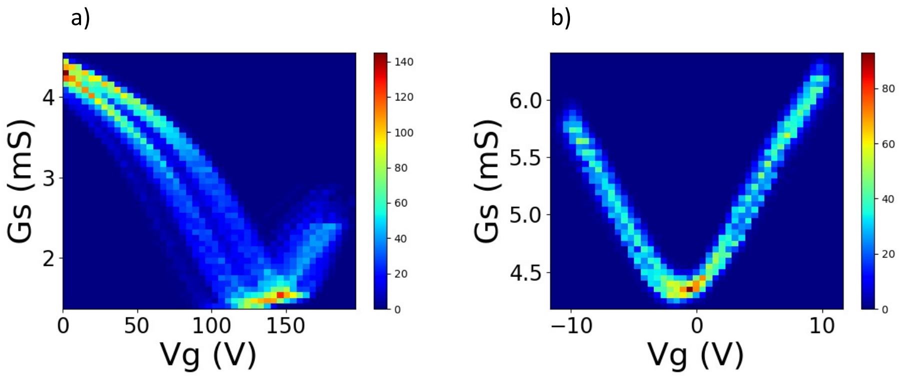

Supplementary Figure 16. Electrical characerization of (a) GFET and **(b)** m-GFET. Colormap represents counts.

We observe the tunning of Dirac point by up to 100-150 V by the electrode decoration. Additionally, different gate voltage ranges generate changes in the number of charge trapped at

the SiOx//Gr interface and subsequently false doped states shifting the Dirac point. In Supplementary Table 2 we summarized the calculated number of charges trapped for experiments displayed in Figure 4b. Trapped charges are calculated with equation 8 in Methods.

Supplementary Table 2. Total of trapped charged.

V_{BG} (V)	V_{BG} (V)	N (cm^{-2})
± 10	~ 0	~ 0
± 50	15	5.4 $x10^{11}$
± 75	34	1.2 x 10^{11}
± 100	55	2.0 x 10^{12}

Actually, this feature varies with the solvent (H_2O, EtOH or acetone) employed before the electrical measurements (Supplementary Figure 17). It indicates that operating these m-GFETs in transient states may discern solvent effect for biosensing applications[18,19].

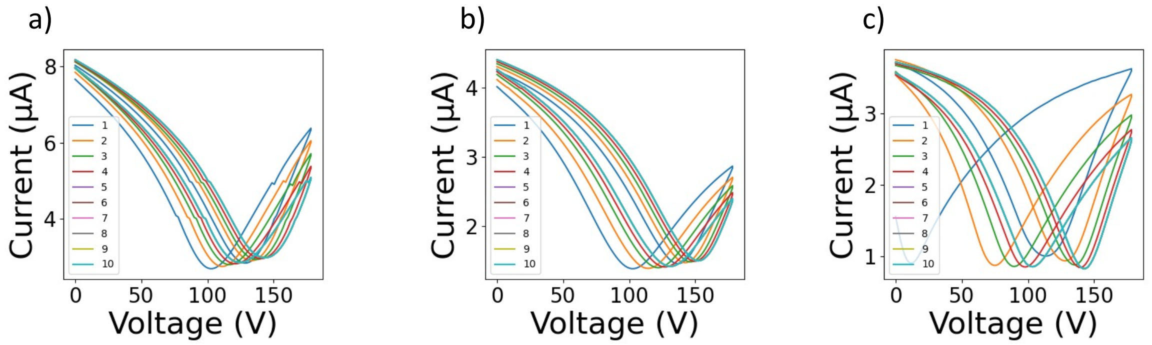

Supplementary Figure 17. Transport characteristics of the GFETs. a) After rinsing with Acetone. **b)** IPA **c)** H_2O.

Although back-gated devices suffer from large parasitic capacitances due to the defects in the SiO_2 layer they are helpful for proof-of-concept purposes. These effects may be exploited for memristive and unconventional computing[20–22] and were employed before for electrical measurements with potential application in biosensing[18,19]. In fact, the motion of molecular entities around topological defects is a key aspect for the rational design of molecular devices[23]. Supplementary Figure 18 illustrates the dynamics of the transport characteristics for a gate voltage of V_{BG} = 10V during 10s and consequent off gate voltage of V_{BG} = 0V during 5s. Along the 7 cycles we found two different processes with τ =3 and 16 s. These memory effects are most probably associated with ions movements depends on the history in each device and can

be deeply investigated and tuned. Static measurements are affected by the charge movement in the system.

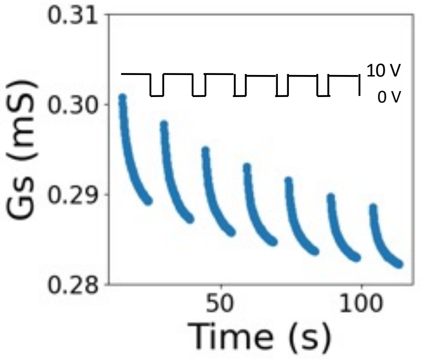

Supplementary Figure 18. Source to drain conductance vs time for a V_{BG} square wave.

For instance, V_{BG} step voltages applied during some time can tune the Dirac Point as it is shown in Supplementary Figure 17.

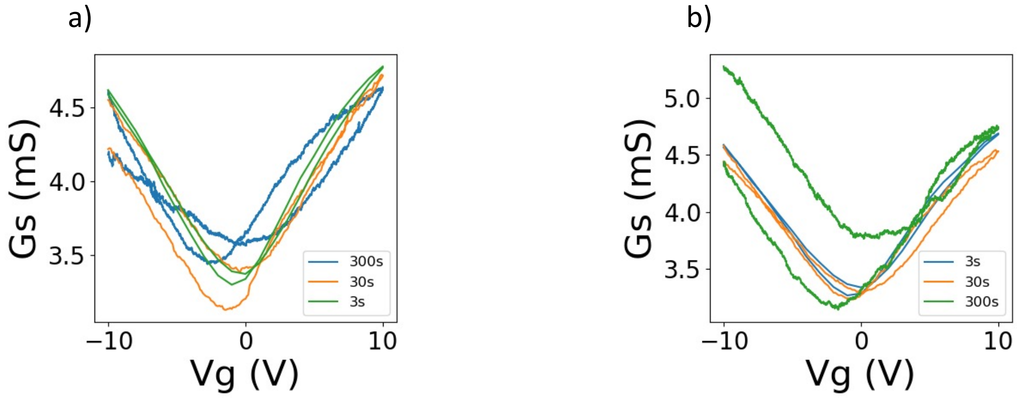

Supplementary Figure 19. Transport characteristics of the m-GFETs as a function of scan rate and direction. The forward scan goes from V_{BG} = -10 V to V_{BG} = 10 V while the backwards foes from V_{BG} = 10 V to V_{BG} = -10 V. **(a)** Sequence going from a fast scan (3 s) to medium (30 s) and slow (300 s). **(b)** Sequence going from slow, medium and fast.

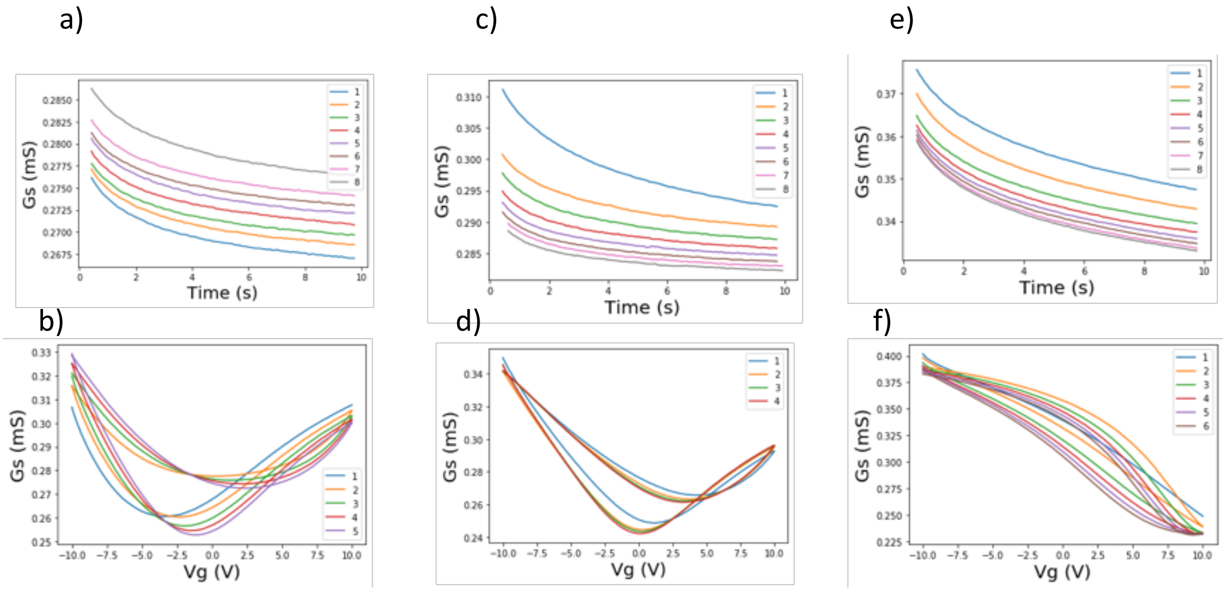

Supplementary Figure 20. Transport characteristics of the m-GFETs, charging effect. (a) Sampling source conductance during 10 s, 8 times at $V_{BG} = 0$ V. **(b)** Subsequent V_{BG} sweeps from $V_{BG} = -10$ V to $V_{BG} = 10$ V. **(c)** Sampling source conductance during 10 s, 8 times at $V_{BG} = 10$ V **(d)** Subsequent V_{BG} sweeps from $V_{BG} = -10$ V to $V_{BG} = 10$ V. **(e)** Sampling source conductance during 10 s, 8 times at $V_{BG} = 50$ V **(f)** Subsequent V_{BG} sweeps from $V_{BG} = -10$ V to $V_{BG} = 10$ V.

Regardless the appearance under the optical microscope. The electrical operation of the device is validated via the source to drain current versus the gate voltage. The position of the Dirac point is used to confirm their proper operation. Considering that the molecular functionalization of the device tunes the V_{DP} to neutrality by changing the working function of the electrode, we assume working devices those with a V_{DP} close to neutrality. Alternatively, samples with a V_{DP} > 30 V are assumed to be defective. As we presented in Supplementary Figure 21, 92% of the fabricated device shift the V_{DP} to neutrality”

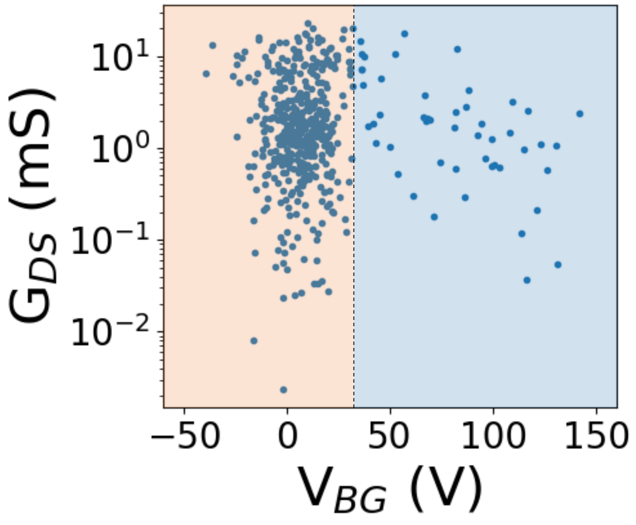

Supplementary Figure 21. Dirac point position, G_{DS} vs. V_{BG} of the 500 m-GFETs.

Demonstration of a logic function with only electrical signals can be achieved with both GFETs and mG-FETs. Here, we select results from a m-GFET curves due to the well centered Dirac point close to $V_{BG} \approx 0V$. In Supplementary Figure 22 there are two curves of I_{SD} vs. V_{BG} for 10 mV and 12mV source to drain voltages.

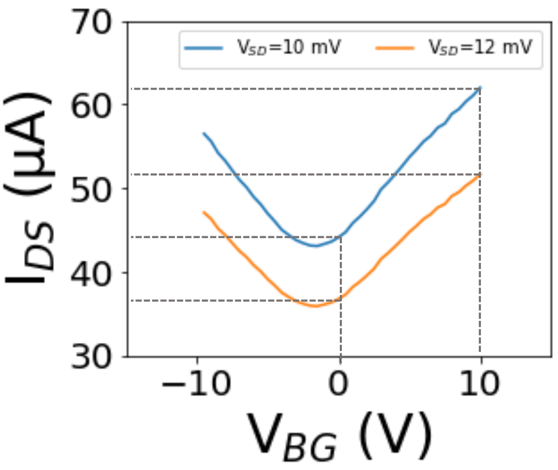

Supplementary Figure 22. Electrical signals of a m-GFET with V_{SD}=10mV and V_{SD}=12mV.

If we consider input 1 the V_{BG} with a value 0V for a digital value "0" and 10V for a digital value of "1" and input 2 with a magnitude of 10 mV for a digital value of "0" and 12mV for a "1" we obtain the following Supplementary Table 3. Therefore, by selecting a threshold of 55 µA (higher than 52 µA and lower than 62 µA) we can have a "and" function or choose a threshold close to 40 µA (higher than 37 µA and lower than 45 µA) getting an "or" function utilizing only electrical inputs.

Supplementary Table 3. Operational values for electrical logic gates.

Input 1: V_{BG}	V_{BG} (mV)	Input 2: V_{SD}	V_{SD} (V)	Output: I_{SD} (μA)
0	0	0	10	37
1	10	0	10	45
0	0	1	12	52
1	10	1	12	62

Supplementary Note 8. m-GFET electrical configuration

The total resistance through the entire junction in the m-GFET devices is the sum of the resistance of one molecular diode in forward direction, the resistance of the graphene and the resistance of the other molecular diode at the reverse bias ($R_T = R_{Fc,F} + R_G + R_{Fc,R}$).

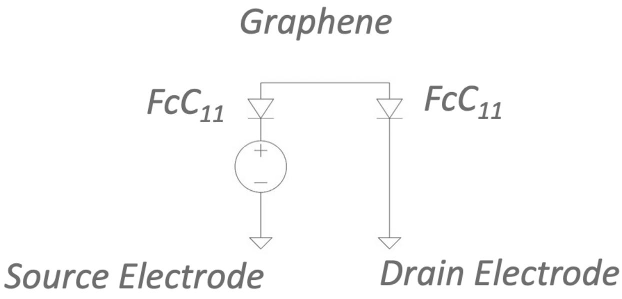

Supplementary Figure 23. Face to face diode arrangement.

Taking into account the band diagram for the face-to-face diode arrangement there should be a window for source to drain bias higher than 3V (η ≈ 0.7) where both diodes operate at forward regime.

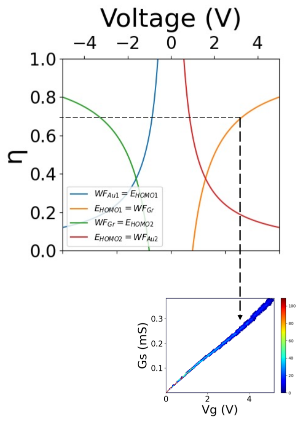

Supplementary Figure 24. (a) Simulated voltage drop as a function of applied voltage. **(b)** 2D source to drain current histogram.

Supplementary Note 9. Ultraviolet photoelectron spectroscopy, X-ray absorption spectroscopy, threshold Photoemission electron microscopy.

Supplementary Figure 25 shows a XAS image acquired at the Fe L3 edge. A clear bright signal corresponding to a higher number of emitted secondary electrons was observed during all the experiments in the region of the electrode marked with a circle. XAS spectra extracted from this region are showed in Supplementary Figure 25a. In each spectrum, two clear contributions can be distinguished coming from the two molecular states, at 708.8 eV for the Fe^{2+} and 710 eV for the Fe^{3+}. In-operando XAS spectroscopy was performed by applying systematically the sequence of 0, 1, 2, 0, -1, -2 V between the source and drain electrodes.

a) b)

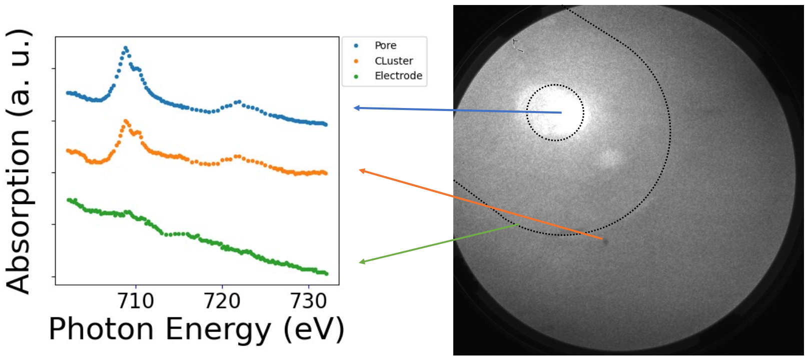

Supplementary Figure 25. Evolution of the X-ray absorption spectra. (a) Local XAS spectra integrated in the circle where the $FcC_{11}SH$ molecules are assembled. **(b)** XAS-PEEM micrograph of the m-GFET electrode at the photon energy Fe L3 edge at room temperature. Field of view is 20 µm.

Supplementary Figure 26 shows the threshold PEEM measurements acquired as a function of the photo-electron energy. The take-off or kinetic energy is controlled by the applied start voltage and it represents the difference between photo-electron energy referenced to the Fermi level, $E - E_F$ and the work function[24]. Then for each pixel we have a map of this minimum thermodynamic work required to remove an electron from the solid to a point in the vacuum. When applying a voltage, we have an operando surface potential in our device. In Supplementary Figure 26a, we observe a m-GFET under the PEEM microscopy and the selected points where start voltage spectra are taken for the different polarization bias. In Supplementary Figure 26b, c and d we observe the start voltage spectra for each point at 0, 1 and -1 voltages respectively. We can notice how spectra are shifted, this shift are related with the voltage drop, for example, between two points.

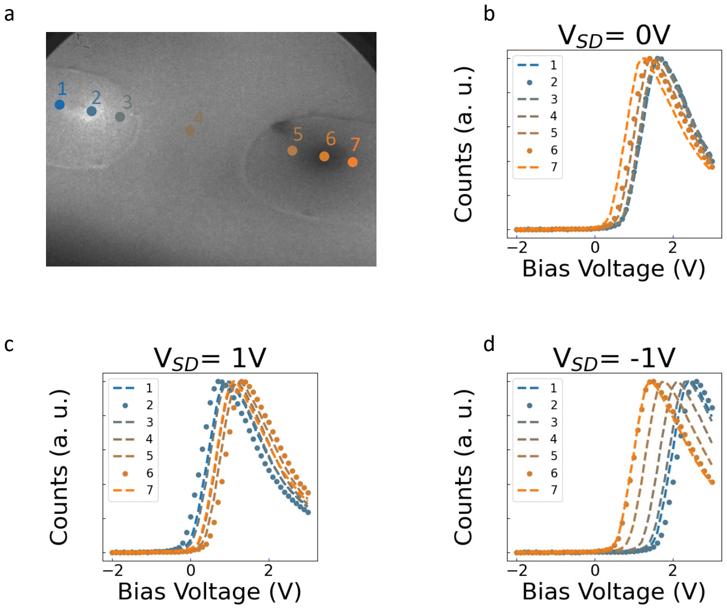

Supplementary Figure 26. Threshold PEEM. (a) mG-FET optical micrograph, **(b)** th-PEEM spectra at $V_{SD} = 0$ V **(c)** th-PEEM spectra at $V_{SD} = 1$ V **(d)** th-PEEM spectra at $V_{SD} = -1$ V. Field of view is 50 µm. Photon energy 702 eV.

Supplementary Note 10. Photo response

Note that the ferrocene redox couple has been used for high-efficiency dye-sensitized solar cells[25] due to differences between the electron and hole transfer times (ps and ns respectively)[26]. In contrast to ionic liquid configuration[27], the backgated joint with the graphene transparency permit not only operando characterization but also using light as an extra gateway. External stimuli access molecular functionality of the device[28]. This ambipolar conductivity based on the ferrocenium stability[29] can be understood in terms of ferrocene based redox switch[30]. Contrary to the m-GFET devices, GFETs do not show photo response as we can note in Supplementary Figure 27.

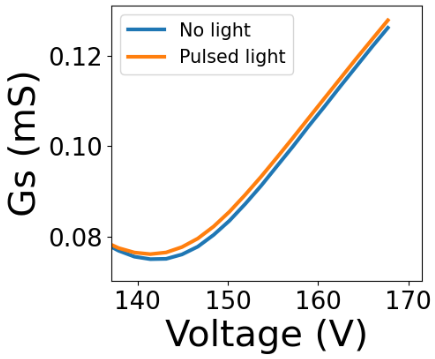

Supplementary Figure 27. Photoresponse. Drain to source conductance at dark and light pulsing conditions for a GFET. Light power: 8.8 mW, $V_{DS} = 0.1V$.

Photo response in m-GFETs depends on the V_{GB}, light power and time as shown in Figures 5d, 5e and 5f in the main text and here in the Supplementary Figure 28.

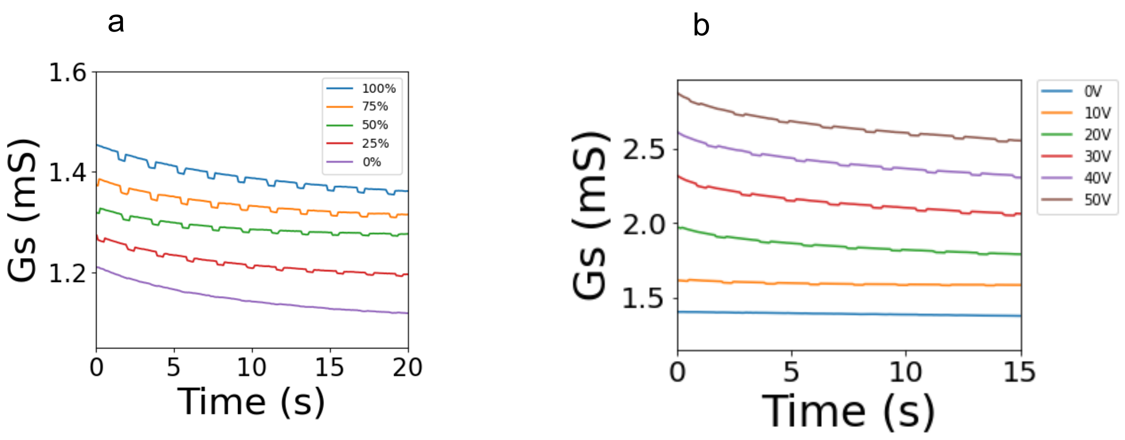

Supplementary Figure 28. Photoresponse. (a) Drain to source conductance as a function of light power (max. 8.8 mW). $V_{GB} = 20V$, $V_{DS} = 0.1V$. **(b)** Drain to source conductance as a function of V_{BG}. Light power: 8.8 mW, $V_{DS} = 0.1V$.

Supplementary References

1. Park, S., Park, J. H., Hwang, S. & Kwak, J. Programmable Electrochemical Rectifier Based on a Thin-Layer Cell. *ACS Appl Mater Interfaces* **9**, 20955–20962 (2017).
2. Nerngchamnong, N. *et al.* The role of van der Waals forces in the performance of molecular diodes. *Nat Nanotechnol* **8**, 113–118 (2013).
3. Tian, H., Dai, Y., Shao, H. & Yu, H. Modulated Intermolecular Interactions in Ferrocenylalkanethiolate Self-Assembled Monolayers on Gold. (2013) doi:10.1021/jp310012v.
4. Reuter, M. G., Hersam, M. C., Seideman, T. & Ratner, M. A. Signatures of cooperative effects and transport mechanisms in conductance histograms. *Nano Lett* **12**, 2243–2248 (2012).
5. Trasobares, J. *et al.* Estimation of π-π Electronic Couplings from Current Measurements. *Nano Lett* **17**, 3215–3224 (2017).
6. Nerngchamnong, N. *et al.* Nonideal Electrochemical Behavior of Ferrocenyl-Alkanethiolate SAMs Maps the Microenvironment of the Redox Unit. *Journal of Physical Chemistry C* **119**, 21978–21991 (2015).
7. D'Andrade, B. W. *et al.* Relationship between the ionization and oxidation potentials of molecular organic semiconductors. *Org Electron* **6**, 11–20 (2005).
8. Heimel, G., Romaner, L., Zojer, E. & Bredas, J. L. The interface energetics of self-assembled monolayers on metals. *Acc Chem Res* **41**, 721–729 (2008).
9. Alévêque, O. *et al.* Electroactive self-assembled monolayers: Laviron's interaction model extended to non-random distribution of redox centers. *Electrochem commun* **12**, 1462–1466 (2010).
10. Laviron, E. Surface linear potential sweep voltammetry. Equation of the peaks for a reversible reaction when interactions between the adsorbed molecules are taken into account. *Journal of Electroanalytical Chemistry* **52**, 395–402 (1974).
11. Umaña, M., Rolison, D. R., Nowak, R., Daum, P. & Murray, R. W. X-ray photoelectron spectroscopy of metal, metal oxide, and carbon electrode surfaces chemically modified with ferrocene and ferricenium. *Surf Sci* **101**, 295–309 (1980).
12. De Leo, L. P. M., De La Llave, E., Scherlis, D. & Williams, F. J. Molecular and electronic structure of electroactive self-assembled monolayers. *Journal of Chemical Physics* **138**, 114707 (2013).
13. Rabti, A., Mayorga-martinez, C. C., Baptista-pires, L., Raoua, N. & Merkoçi, A. Ferrocene-functionalized graphene electrode for biosensing applications. *Analytica Chimica Acta journal* **926**, 28–35 (2016).
14. Watcharinyanon, S., Moons, E. & Johansson, L. S. O. Mixed self-assembled monolayers of ferrocene-terminated and unsubstituted alkanethiols on gold: Surface structure and work function. *Journal of Physical Chemistry C* **113**, 1972–1979 (2009).
15. Pirkle, A. *et al.* The effect of chemical residues on the physical and electrical properties of chemical vapor deposited graphene transferred to SiO2. *Appl Phys Lett* **99**, 2009–2012 (2011).
16. Song, P. *et al.* Stable Molecular Diodes Based on π–π Interactions of the Molecular Frontier Orbitals with Graphene Electrodes. *Advanced Materials* **30**, 1–8 (2018).
17. Chaves, F. A., Jiménez, D., Cummings, A. W. & Roche, S. Physical model of the contact resistivity of metal-graphene junctions. *J Appl Phys* **115**, (2014).
18. Dai, X. *et al.* Modularized Field-Effect Transistor Biosensors. *Nano Lett* **19**, 6658–6664 (2019).
19. Ono, T. *et al.* Electrical Biosensing at Physiological Ionic Strength Using Graphene Field-Effect Transistor in Femtoliter Microdroplet. *Nano Lett* **19**, 4004–4009 (2019).

20. Schranghamer, T. F., Oberoi, A. & Das, S. Graphene memristive synapses for high precision neuromorphic computing. *Nat Commun* **11**, 1–11 (2020).
21. Burzurí, E., Granados, D. & Pérez, E. M. Physically Unclonable Functions Based on Single-Walled Carbon Nanotubes: A Scalable and Inexpensive Method toward Unique Identifiers. *ACS Appl Nano Mater* **2**, 1796–1801 (2019).
22. Viero, Y. *et al.* Light-Stimulatable Molecules/Nanoparticles Networks for Switchable Logical Functions and Reservoir Computing. *Adv Funct Mater* **28**, 1801506 (1–10) (2018).
23. Nirmalraj, P., Daly, R., Martin, N. & Thompson, D. Motion of Fullerenes around Topological Defects on Metals: Implications for the Progress of Molecular Scale Devices. *ACS Appl Mater Interfaces* **9**, 7897–7902 (2017).
24. Lavayssière, M., Escher, M., Renault, O., Mariolle, D. & Barrett, N. Electrical and physical topography in energy-filtered photoelectron emission microscopy of two-dimensional silicon pn junctions. *J Electron Spectros Relat Phenomena* **186**, 30–38 (2013).
25. Daeneke, T. *et al.* High-efficiency dye-sensitized solar cells with ferrocene-based electrolytes. *Nat Chem* **3**, 211–215 (2011).
26. Ponseca, C. S., Chábera, P., Uhlig, J., Persson, P. & Sundström, V. Ultrafast Electron Dynamics in Solar Energy Conversion. *Chem Rev* **117**, 10940–11024 (2017).
27. Jia, C. *et al.* Quantum interference mediated vertical molecular tunneling transistors. *Sci Adv* **4**, eaat8237 (2018).
28. Xin, N. *et al.* Concepts in the design and engineering of single-molecule electronic devices. *Nature Reviews Physics* **1**, 211–230 (2019).
29. Tarafder, K., Surendranath, Y., Olshansky, J. H., Alivisatos, A. P. & Wang, L. W. Hole transfer dynamics from a CdSe/CdS quantum rod to a tethered ferrocene derivative. *J Am Chem Soc* **136**, 5121–5131 (2014).
30. Fabbrizzi, L. The ferrocenium/ferrocene couple: a versatile redox switch. *ChemTexts* **6**, 1–20 (2020).
